# Onions and Bubbles: Models of the Social Determinants of Health

**Published:** 2007-09-15

**Authors:** Lynne S. Wilcox

The discussion of society and health is complex and sometimes confusing. What is social medicine? What is community medicine? What is the socioecologic model? All these terms have been used to describe the relationship between health and other social conditions. Even public health professionals may find the differences blurred.

The previous issue of *Preventing Chronic Disease* discussed community health and community-based participatory research (1). Multiple factors affect a community's function and, in turn, the health of its citizens, and our October issue examines the broader context in which communities operate. For this issue, we welcome Marilyn Metzler of McKing Consulting as our guest editor.

In 2005 the World Health Organization (WHO) established the Commission on Social Determinants of Health, which identified nine areas of concentration: early child development, globalization, health systems, urban settings, women and gender equity, social exclusion, employment conditions, priority public health conditions, and measurement and evidence (2).

A generous range of models is available to explain the impact of these factors on an individual's health. Some models resemble onions — concentric circles of variables, each construed as operating at a more distal position from the individual (3). One group provided an inverted example of the pyramid (4). The causal web (5) is another representation. These images imply linear, if bidirectional, relationships operating in two dimensions.

Yet we know the true relationships are more complex. A visual model might be more meaningful if considered in three or more dimensions. Glass and McAtee observe that another image is that of a running stream, again suggesting "upstream," "distal" factors that affect "downstream," "proximate" factors. Their concepts offer a three-dimensional model that uses the axes of time and biological-social organization (6).

Now consider the model of a cascade of soap bubbles, with the individual bubble existing among many in a cluster. A single bubble interfaces with many others, and if one bubble pops, the surface tension and connectivity of the others change throughout the cascade (7). The cascade's properties are dynamic: the bubbles merge and increase or decrease in size and shape in relation to one another. If air blows across the entire cascade or the water flow changes, all the bubbles may be affected and may perhaps even disappear.

Then think of the cluster of bubbles as the collection of all factors affecting health: environment, working conditions, economy, education, culture, and health systems. These influences affect the individual in both direct and indirect fashion, just as a bubble is influenced directly by a companion bubble's interface but also indirectly through the companion bubble's connections to other surfaces.

This analogy suggests that for an individual citizen, factors may operate not only through a hierarchy such as community–state–federal but also directly on the individual. The federally sponsored Medicare program, for example, provides funds for direct health care without passing through community review. The diet of an immigrant child may be more heavily affected by attitudes in his parents' country of origin than by practices in his new, local culture. Employment conditions may be more directly influenced by business decisions in a company headquarters 500 miles away than by local employee concerns.

Another implication of this model is that not all factors are focused on the individual or community. If the destructive winds of an economic depression or widespread war blow across the cascade, all systems will change, and the individual will be caught up in these forces rather than be their focus. The cascade properties also illustrate the unintended consequences that may result from social policy interventions.

This concept is not new, only another attempt to explain the forces we all recognize. So why are we in the United States so fond of models focused on the individual? Porter summarizes aspects of American and British medical history that led this country away from the more society-based concepts of medicine and health that arose in Europe and elsewhere in the 20th century (8). By mid-century, U.S. life insurance companies had already identified relationships between lifestyle, overweight, and cardiac disease. The Framingham study was initiated in the 1940s to examine individual behavior and track its connection to coronary heart disease over time. Doll and Hill published their findings on cigarette use and lung cancer in 1950. The relationship between exercise and obesity was also identified, and by the 1950s, medical interest in the health effects of overweight was strong.

These discoveries pointed to individual experience, and it is not surprising that health promotion models also focused on individual responses and behaviors. Furthermore, this concept appealed to the deeply held American value of self-determination. The United States is primarily populated by the descendents of immigrants who uprooted their lives because they believed that individuals had the capacity to change their circumstances. It followed that sufficiently self-disciplined citizens should be able to control their own behaviors. Thus our common models center on the individual and suggest that other forces are secondary.

But this laudatory value, so successful in establishing a new democracy in the 18th century, is not well suited to protecting the public's health in the 21st century. Articles in this issue explore the multiple social interfaces that affect health. Referring to the WHO list of concentration areas, for *early child development*, this issue discusses a program for encouraging home-based nutrition programs for preschool American Indian children (9). *Health systems* studies include examining the impact of alternative mammogram outreach programs on Latina women with different types of insurance (10), National Health Interview Survey data on barriers to cervical cancer screening (11), physician advice to people with disabilities on smoking cessation (12), repeat mammography for low-income women (13), and educational toolboxes to enable *promotores* to address mental health issues for their diabetes patients (14).

Regarding *urban settings*, we have a report on smoke-free zones in public parks (15), but we also have a report on indoor air issues in rural settings (16). Kumanyika and colleagues provide an excellent discussion of the links between obesity and *social exclusion* among African Americans, especially women, drawing a synthesis of insights from family sociology, literature, philosophy, transcultural psychology, marketing, economics, and the built environment (17). Bopp and colleagues describe a physical activity promotion model that was disseminated through South Carolina African Methodist Episcopal churches (18). Hill and colleagues describe five years of community coalition experience along the U.S.–Mexico border (19). *Employment* and socioeconomic conditions are examined as they affect binge drinking by occupational status in North Dakota (20) and the direct relationship between family income and mammography screening in Hawaii (21). Braveman (22) provides an extensive discussion of the impact of poverty in the United States on the health of its citizens.

Appropriately, this issue's strongest showing is in *measurement and evidence*. Van Duyn and colleagues introduce four articles on the role of society in energy balance programs — programs that encourage a healthy balance between calories consumed and calories expended — for Native Hawaiians and African Americans and for Hmong and Latina populations. (23-27). Ham and colleagues examine data from four national surveys to assess physical activities in multiple Hispanic populations (28). Metzler reviews several reports on the indicators and determinants of community health status (29).

It is nearly impossible to visualize the "bubbles" for all the areas identified by WHO. Public health is not the entire cascade, and our field will not have the lead on addressing all social determinants. We have a long road ahead. But even the simple effort to model these interfaces is a step forward.
